# Specificity vs. Synergy Between Single-Strain and Multi-Strain Probiotics for Ulcerative Colitis Treatment: A Review of the Literature

**DOI:** 10.3390/biomedicines14061386

**Published:** 2026-06-19

**Authors:** Muhammad Ikhmal Rosali, Dinesh Prasad V. Thanga Velu, Mohd Helmy Mokhtar, Raja Affendi Raja Ali, Norfilza Mohd Mokhtar, Adila A. Hamid

**Affiliations:** 1 Department of Physiology, Faculty of Medicine, Universiti Kebangsaan Malaysia, Kuala Lumpur 56000, Malaysia; p145048@siswa.ukm.edu.my (M.I.R.); helmy@ukm.edu.my (M.H.M.); 2 Department of Tissue Engineering and Regenerative Medicine, Faculty of Medicine, Universiti Kebangsaan Malaysia, Kuala Lumpur 56000, Malaysia; p153533@siswa.ukm.edu.my; 3 Department of Biomedical Sciences, Jeffrey Cheah Sunway Medical School, Faculty of Medical and Life Sciences, Sunway University, Sunway City 47500, Malaysia; affendi@sunway.edu.my; 4 GUT Research Group, Faculty of Medicine, Universiti Kebangsaan Malaysia, Kuala Lumpur 56000, Malaysia; 5 International Medical School, Management & Science University, Shah Alam 40000, Malaysia; norfilza@msu.edu.my

**Keywords:** probiotics, strain specificity, synergistic mechanism, ulcerative colitis

## Abstract

Ulcerative colitis (UC) is a chronic disease marked by mucosal inflammation of the colon, and its prevalence has progressively increased worldwide. Gut dysbiosis is recognized as a key contributor to its pathogenesis. Although conventional treatments are effective in managing symptoms, they often fail to address the underlying gut microbial imbalance, prompting growing interest in microbiota-based therapies. Probiotic supplementation has demonstrated potential to modulate the disease. However, its clinical application is limited by variability in formulations and strain composition. Debate persists regarding the relative benefits of single-strain probiotics (SSPs), which depend on strain specificity, versus multi-strain probiotics (MSPs), which may provide synergistic effects. The literature remains inconclusive, with some studies indicating that MSPs outperform SSPs, while others emphasize the importance of strain specificity. This review describes the mechanistic basis of both approaches and descriptively synthesizes their clinical efficacy in UC management based on the clinical studies published between 2018 and 2025. Several studies report that both SSPs and MSPs are associated with clinical improvements, including reduced disease activity, symptom alleviation, and enhanced endoscopic outcomes. Given the methodological heterogeneity across included studies, comparative findings should be interpreted with appropriate caution. A direct head-to-head trial could provide a better understanding to determine the optimal approach. Advancing toward personalized probiotic therapy may further enhance the clinical application of probiotics for disease management.

## 1. Introduction

Ulcerative colitis (UC) is a subtype of inflammatory bowel disease (IBD) that affects the mucosal lining of the large intestine and is characterized by persistent, continuous inflammation [1]. The prevalence of UC has been predominantly high in Western regions, evolving into a phase of compounding prevalence with low incidence but high prevalence rates, compared to emerging industrialized countries in Asia, Africa, and South America, which are experiencing an acceleration phase with higher incidence rates [2]. Epidemiologically, the global incidence of UC has been estimated at 1 in 200 individuals, with projections indicating continued growth in prevalence through 2030 [3]. This pattern has raised a major public health concern, with an escalating burden of disease and mortality among UC patients [4]. The precise cause of UC remains elusive, with multiple etiologies including genetic susceptibility, dysregulated mucosal and systemic immune responses, environmental triggers, and microbial shifts in the composition and function of the intestinal microbiota [5]. These shifts, known as gut dysbiosis, result in an imbalance of microbial communities characterized by a decrease in beneficial species (e.g., *Faecalibacterium prausnitzii*, *Roseburia*) and an increase in pathogenic species (e.g., *Escherichia coli*, *Enterococcus faecium*, *Ruminococcus gnavus*), leading to a loss of microbial diversity, reduced short-chain fatty acid production, and elevated inflammatory response [6,7]. As a result, epithelial barrier integrity is disrupted, contributing to a state of ‘leaky gut’ with increased intestinal permeability, allowing the translocation of pathogens, their toxins, and other potentially harmful substances into the internal environment [8,9].

Disease severity was categorized as mild, moderate, or severe, measured using various indices, including symptoms, endoscopy, and histology, reflecting the degree of inflammation in UC patients and indicating the need for immediate medical intervention [10]. Conventional treatments such as 5-aminosalicylates (5-ASAs), corticosteroids, immunosuppressants, biologics, and small molecules have been used to induce and maintain remission by modulating the immune system and reducing inflammation [11]. However, these interventions may not effectively resolve the underlying dysbiosis, leading to primary non-response or secondary loss of response, necessitating the development of gut microbiota-derived treatments to restore microbial homeostasis [12]. One available treatment is probiotics, defined as live microorganisms that confer beneficial effects on the host when administered in adequate amounts. They restore microbial balance by modulating immune responses, inhibiting pathogen colonization, strengthening the gut epithelial barrier, and facilitating the production of beneficial metabolites [13,14,15]. Therefore, the use of probiotics as an alternative therapy may enhance disease management in UC. However, the expected health benefits of probiotics can vary significantly depending on the administered dosage, survivability in the digestive tract, and strain composition [16,17,18]. Regarding strain composition, probiotics can be categorized into two types: single-strain probiotics (SSPs), which refer to an individual strain aimed at a specific therapeutic target, and multi-strain probiotics (MSPs), which are mixtures of two or more strains that work synergistically to provide broader health benefits.

In recent years, interest has increased in exploring the efficacy of SSPs and MSPs, particularly regarding their mechanisms of action in disease treatment. For example, McFarland et al. [19] systematically reviewed the efficacy of SSPs and MSPs across 65 randomized clinical trials (RCTs) on various diseases and hypothesized that probiotic efficacy is strictly strain- and disease-specific. A prospective observational study of clinicians’ probiotic prescription patterns for pediatric patients found a preference for single-strain probiotics [20]. Another study observed that probiotic mixtures appear effective across a wide range of endpoints, showing greater efficacy than single strains, although the beneficial effects remain unclear, possibly due to interactions between strains or to the use of higher doses in some studies [21]. This raises a critical question in modern UC management: does its complex pathogenicity require the specificity of a targeted single strain, or the synergy of a multi-strain probiotic? Therefore, this review serves a dual purpose: to evaluate the specificity and synergistic mechanisms of single-strain and multi-strain probiotic therapy, and to descriptively assess clinical evidence from human studies examining their efficacy in managing UC, based on studies published between 2018 and 2025.

## 2. Methods

A comprehensive literature search was conducted for this narrative review to identify relevant studies using SSPs or MSPs in the management of UC. The search was performed across multiple databases, including PubMed, Scopus, Ovid MEDLINE, Web of Science, and Google Scholar. A combination of search terms was used: ‘ulcerative colitis’ AND ‘probiotics’ AND (‘synergy’ OR ‘specificity’ OR ‘single-strain’ OR ‘multi-strain’). Only human clinical studies published between 2018 and 2025 were included, with no language or region-of-origin restrictions. No restriction was imposed on study methodology (e.g., randomized controlled trials, retrospective studies, open-label trials) or on the endpoints measured. This review describes the mechanistic specificity of SSPs and the synergistic interactions of MSPs in the treatment of UC, while providing a descriptive evaluation of clinical evidence from human studies reporting the efficacy of both probiotic approaches in UC management. Owing to the narrative nature of this review, the findings are synthesized descriptively rather than statistically, and the variability across included studies is acknowledged as an inherent limitation.

**Figure 1:**
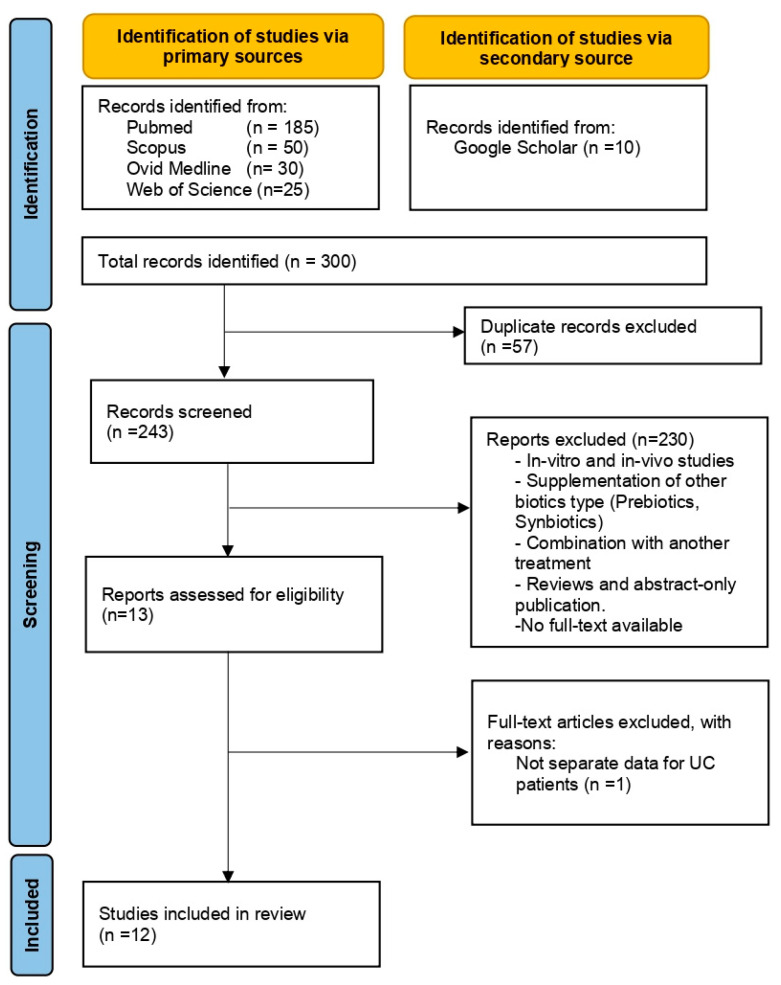
PRISMA flowchart of the structured literature search for the narrative review.

The retrieved references were imported into EndNote (version 20) for data organization and deduplication. The remaining articles then underwent a two-stage screening process conducted by the authors: (1) title and abstract screening, followed by (2) full-text assessment to determine eligibility for inclusion in the narrative review. In total, 300 studies were initially identified, of which 57 were duplicates. The remaining 243 studies were screened, and eventually 12 studies were included. As this is a narrative review, strict adherence to the PRISMA protocol, typically applied to systematic reviews, was not required. However, a PRISMA flowchart is presented in Figure 1 to provide an overview of the search and selection strategy applied.

## 3. Gut Dysbiosis in Ulcerative Colitis

UC is strongly associated with gut dysbiosis, resulting in reduced microbial diversity and significant shifts in mucosal and luminal communities [22]. Gut dysbiosis in UC is marked by the depletion of beneficial taxa such as *Bifidobacterium* and key short-chain fatty acid (SCFA)-producing bacteria, including *Faecalibacterium prausnitzii*, *Roseburia intestinalis*, and *Eubacterium rectale* [23,24]. Some studies have also reported taxonomic shifts within *Bifidobacteria*, with *Bifidobacterium adolescentis* being more prevalent in healthy mucosa, while *Bifidobacterium angulatum* predominates in UC [25]. At the same time, opportunistic pathogens such as adherent-invasive *Escherichia coli*, *Candida albicans*, and *Ruminococcus gnavus* become enriched, promoting inflammatory pathways, epithelial adhesion, and cytokine release [23,24,26]. Moreover, gut microbiota may form clustered microcolonies rather than diffuse distributions, intensifying localized antigen exposure and inflammatory damage [27]. These microbial alterations contribute to a self-reinforcing cycle in which reduced SCFA production, increased oxidative stress, and impaired epithelial barrier function perpetuate chronic inflammation [28,29,30].

## 4. Specificity Effects of Single-Strain Probiotics on Ulcerative Colitis Patients

**Figure 2:**
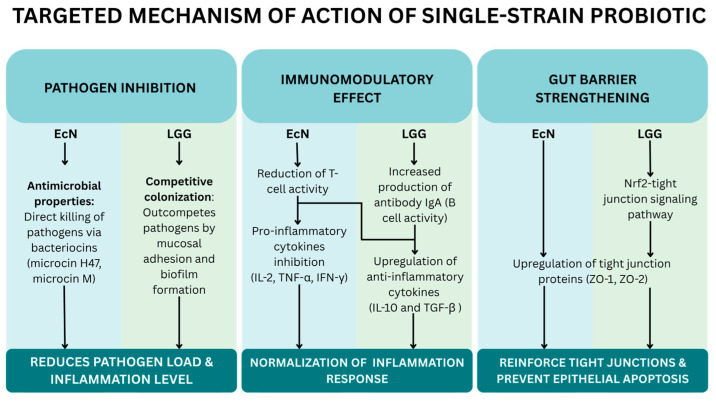
Comparative mechanisms of action of single-strain probiotics (SSPs), *Escherichia coli* Nissle 1917 (EcN), and *Lactobacillus rhamnosus* GG (LGG). The schematic illustrates three primary pathways of action. (1) Pathogen inhibition via bacteriocin production (microcin H47, microcin M) and competitive colonization via mucosal adhesion and biofilm formation. (2) Immunomodulation through the suppression of pro-inflammatory cytokines such as interleukin (IL-2), tumor necrosis factor-alpha (TNF-α), and interferon-gamma (IFN-γ) and upregulation of anti-inflammatory markers such as interleukin-10 (IL-10) and Transforming Growth Factor-beta (TGF-β). (3) Strengthening of the intestinal barrier by upregulating tight junction proteins, Zonula Occludens-1 (ZO-1) and Zonula Occludens-2 (ZO-2) through Nrf2 signaling.

Single-strain probiotics (SSPs) are used for targeted biological intervention, employing specific bacterial strains that have been extensively studied for their therapeutic properties. Notable SSPs include *Escherichia coli* strain Nissle 1917 (EcN) and *Lactobacillus rhamnosus* GG (LGG), which are commonly used to treat various gastrointestinal disorders. The concept of specificity is based on their ability to modulate the gut microenvironment through distinct mechanisms of action. For example, EcN alleviates intestinal inflammation through multiple modulatory pathways, including direct antimicrobial inhibition of pathogens by producing bacteriocins (microcin H47, microcin M), strengthening the epithelial barrier by upregulating tight junction proteins, such as Zonula Occludens-1 (ZO-1) and Zonula Occludens-2 (ZO-2), and exerting immunomodulatory effects that regulate cytokine balance and T-cell activity [31,32,33]. Similarly, LGG provides therapeutic effects by reinforcing gut barrier integrity through activation of the nuclear factor erythroid 2-related factor 2 (Nrf2) tight junction pathway and upregulation of tight junction proteins, enhancing immune defense by increasing immunoglobulin A (IgA) and interleukin-22 (IL-22) secretion, and inhibiting pathogen growth through competitive colonization through mucosal adhesion and biofilm formation [34,35,36]. These distinct, strain-specific molecular actions support the concept of specificity in probiotic treatment, emphasizing the precise interaction between the strain and the host-disease context, as shown in Figure 2.

Four studies involving 351 participants using single-strain probiotics were included, consisting of two retrospective single-arm studies [37,38] and two randomized clinical trials [39,40]. Participants included patients in remission for maintenance of remission and those with mild to moderate disease for induction of clinical response. The probiotic interventions varied, with two studies using escalating or tapering dose protocols [37,39] and two using a fixed daily dosage [38,40]. Probiotic dosages also differed among the studies, ranging from 2.5 × 10^9^ CFU/day to 5 × 10^9^ CFU/day [37,38,39], while one study used a dual dosage intervention, comparing a single dose of 1.2 × 10^10^ CFU/day to a double dose of 2.4 × 10^10^ CFU/day [40]. Most studies maintained their standardized UC medications, except for one LGG monotherapy interventional study. Study duration ranged from short-term, within four to eight weeks [37,39,40], to long-term, 52 weeks [38]. Table 1 summarizes the clinical studies of SSPs for UC treatment.

In a randomized controlled trial assessing the efficacy of EcN alongside standard treatment in patients with mild to moderate disease, Park et al. (2022) [39] reported that a greater proportion of patients in the EcN-treated group showed a significant clinical response (39.7%) and improvement in quality of life (13.3%) compared to the placebo group (21.7% and 1.7%, respectively) after four weeks of intervention. In addition, an observational single-arm study assessing the efficacy of EcN in patients in remission demonstrated a significant reduction in partial Mayo score (pMS) [38]. These findings highlight the role of EcN in improving symptoms and maintaining remission. Furthermore, the supplementation with EcN in mild to moderate UC patients significantly induced endoscopic remission (46.4%) compared to the control group (21.7%) [39], further supporting the role of EcN in improving the gut epithelial barrier.

In contrast, fecal calprotectin (FC), a biomarker of gut inflammation, showed heterogeneous responses among patients with inactive disease treated with EcN. In remission patients with elevated baseline FC levels, a significant reduction was observed following EcN supplementation [37], whereas patients with low baseline FC levels exhibited no significant change [38]. EcN reduced residual intestinal inflammation when FC levels were elevated during inactive disease and maintained FC at normalized levels, thereby lowering the probability of relapses in a stabilized disease state. Moreover, supplementation with EcN in patients with mild to moderate UC does not restore microbial diversity, highlighting its limited capacity to modulate the gut microenvironment despite significant improvement in clinical and endoscopic responses [39].

Evidence for LGG was limited. A single study assessing LGG as monotherapy in patients with mild to moderate UC previously treated with standard care reported an overall clinical response rate of 58%, with only 4% of patients experiencing disease worsening [40] as assessed with pMS. Both doses showed significant reductions in pMS, with the single dose producing a greater reduction (*p* < 0.001) compared to the double dose (*p* < 0.005). Dose–response analysis revealed no significant differences between low- and high-dose LGG, suggesting that lower doses may be sufficient to achieve clinical benefit. Moreover, a subset analysis (*n* = 27) for endoscopic activity revealed a significant reduction in the Endoscopic Mayo score following LGG intervention. Overall, 26% showed an endoscopic response, 70% remained stable, and 4% experienced a flare-up.

## 5. Synergistic Effects of Multi-Strain Probiotics on Ulcerative Colitis Patients

**Figure 3:**
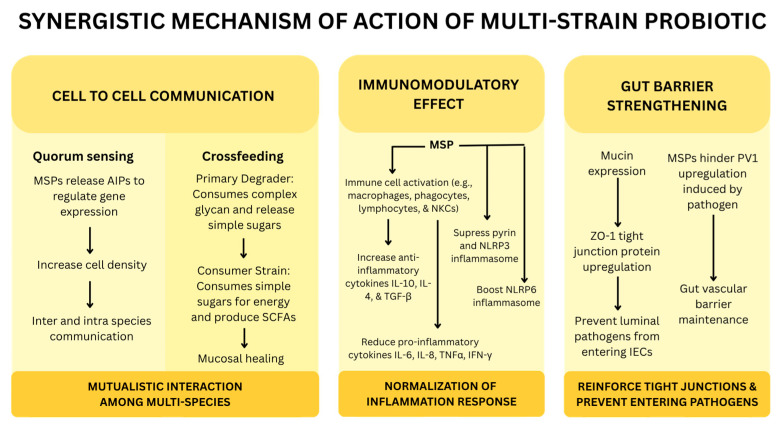
Synergistic mechanisms of MSPs in the regulation of intestinal homeostasis. The schematic illustrates the three main functional actions of MSPs in maintaining gut homeostasis. (1) Cell-to-cell communication; (2) immunomodulatory effects; and (3) gut barrier strengthening.

Multi-strain probiotics (MSPs) are formulations containing two or more strains, commonly combining *Lactobacillus* and *Bifidobacterium* species, both genera known to act synergistically and improve treatment outcomes [41]. The synergistic effects of MSPs are achieved through various mechanisms, including cell–cell communication, interactions with host tissues, and modulation of the immune system (Figure 3) [42]. MSPs enhance cell-to-cell communication via quorum sensing, in which small signaling molecules are easily detected by the cells [43]. Quorum sensing is known to increase cell density and regulate damaged cells in the inflamed colon [44]. Gram-positive bacteria such as *Lactobacillus* spp. release autoinducing peptides that regulate gene expression and environmental factors within intestinal epithelial cells. Synergistic MSPs communicate broadly between inter- and intra-species after cell density reaches a threshold [45].

Furthermore, MSPs exhibit strong adhesion to the mucosa of the digestive tract, as the included strains may complement each other [46]. *Bifidobacteria* strains provide sugars to other beneficial strains, such as *Lactobacillus* species, in the gut through glycan cross-feeding [47]. Complex glycans and dietary fibers in the gut are initially broken down by primary degrader strains, which secrete extracellular glycoside hydrolases to cleave long-chain polysaccharides into smaller oligosaccharides. These released substrates are then utilized by specialist or consumer strains, creating a cooperative metabolic network known as cross-feeding. This process establishes a bidirectional interaction in which the metabolic product of one strain serves as a substrate for another, overcoming the enzymatic limitations of individual strains, as no single probiotic possesses the genetic capacity to degrade every type of complex carbohydrate. Through synergistic growth, co-cultured strains achieve greater metabolic activity, biomass, and survival compared to SSPs. Their collective fermentation activity enhances the production of beneficial SCFAs, which lower intestinal pH, inhibit pathogenic microbes, strengthen the intestinal epithelial barrier, and regulate immune and inflammatory responses. For example, one strain may provide essential nutrients (such as folate, para-aminobenzoic acid (pABA), or vitamins) that support the growth and metabolic output of another strain [48]. These SCFAs produced by microbes reduce inflammation and preserve the gut lining, which is a desired treatment effect in UC [49]. This complementary relationship between strains in MSPs enables them to outgrow pathogenic bacteria by enhancing mucosal adhesion while simultaneously increasing beneficial bacteria (such as *Blautia* and *Collinsella*) [50].

In addition, multiple strains in MSPs can target various binding sites, providing broader mucosal coverage [42]. By occupying mucosal regions, MSPs deprive pathogenic bacteria such as *Prevotella*, *Escherichia-Shigella*, and *Klebsiella* in UC of essential nutrients, leading to their decline and promoting gut eubiosis [50]. Elimination of pathogenic bacteria in the colon occurs not only through competitive exclusion but also through additional mechanisms, such as mucin expression, which upregulates the tight junction protein, ZO-1 [51], and prevents luminal pathogens from entering intestinal epithelial cells [52]. Despite upregulation of tight junction proteins, MSPs maintain gut vascular barrier (GVB) integrity by hindering the upregulation of plasmalemma vesicle-associated protein-1 (PV1) induced by gut pathogenic infections [52].

Moreover, MSPs modulate the immune system through multiple mechanisms, providing broader immunomodulatory coverage than SPPs. In particular, MSPs reduce nuclear factor-kappa-B (NFκB) pathway signaling and inhibit NLRP3 inflammasome activation, resulting in modulation of cytokine production characterized by upregulation of anti-inflammatory cytokines and concurrent downregulation of pro-inflammatory cytokines [53,54]. The immunomodulatory effect of MSPs arises from their interaction with immune cells rather than being solely cytokine-focused. MSPs activate macrophages and phagocytes to engulf pathogenic bacteria [55], while natural killer cells (NKCs) and T-helper 1 (Th1) lymphocytes synthesize interferon-γ (IFN-γ) as part of an immunoregulatory response [56]. For example, MSPs reduce IFN-γ-producing cluster of differentiation 4 (CD4)+ T cells, NKCs, and B cells [53]. In addition, MSPs containing *Lactobacillus* and *Bifidobacterium* strains produce SCFAs that regulate intestinal health by enhancing the NLRP6 inflammasome, which strengthens the gut barrier and limits pathogenic dysbiosis [57,58]. These probiotics simultaneously suppress the hyper-inflammatory sensors Pyrin and NLRP3 through competitive exclusion of toxins, reducing chronic tissue damage and pyroptosis [57,59]. This combined mechanism maintains a stable immune environment by hindering activation of caspase-1, which triggers the three key pro-inflammatory components, which are IL-1β, IL-18, and gasdermin D (GSDMD) [60].

Although there is no direct evidence of MSP inhibiting NLRP3, MSP nanoparticles (containing *Lactobacillus acidophilus*, *Bifidobacterium bifidum*, and *Streptococcus thermophilus*) directly downregulated the expression of the NLRP3 inflammasome, caspase-1, and IL-18 in the colitis model [61]. The suppression of NLRP3 by MSP is driven by SCFA production and mitophagy activation [62]. This action prevents the mitochondrial reactive oxygen species (ROS) spikes that normally trigger NLRP3 assembly, thereby saving the mucosal barrier [63]. In addition, multiple strains in the probiotic provide nutrients to intestinal epithelial cells (IECs), preserve mucin production, and potentially create an environment with reduced immune cell activation, thereby lowering inflammation in UC patients [52,64]. Collectively, MSPs act synergistically to eliminate pathogens and restore gut homeostasis through cross-feeding of beneficial bacteria, quorum sensing, immunomodulatory regulation, and redistribution of tight junction proteins.

Eight studies involving a total of 542 participants investigated MSP supplementation in ulcerative colitis (UC). Five studies assessed the induction of remission in mild to moderate UC [65,66,67,68,69], while three studies focused on inactive disease, investigating maintenance of remission or management of concurrent irritable bowel syndrome (IBS)-like symptoms [70,71,72]. Most studies were randomized clinical trials [65,66,67,68,69,70,71], with an observational single-arm study [72]. Probiotic formulations varied in composition, ranging from low-complexity combinations (two to four strains) to high-complexity consortia (more than five strains). Both types demonstrated similar core mechanisms of action, but their strain complexity determines the level of functional redundancy, defined as the overlapping metabolic roles across bacterial strains. This redundancy provides flexibility for adaptation in the harsh gut microenvironment and is crucial for sustaining their function [73]. Generally, low-complexity MSPs exhibit low functional redundancy due to the limited combination of species, while high-complexity MSPs often cluster multiple species, resulting in elevated functional redundancy that provides a distinct adaptive advantage.

Regarding formulation, five studies used low-complexity multi-strain combinations [65,66,70,71,72], while three studies employed high-strain count combinations [67,68,69]. The compositions primarily consisted of *Lactobacillus* spp. and *Bifidobacterium* spp., with other genera such as *Enterococcus* spp., *Clostridium* spp., and *Streptococcus* spp. also reported. Treatment duration ranged from short-term studies of about four weeks to longer durations of around 16 weeks. Probiotic dosage varied across the studies, ranging from 3 × 10^6^ to 9 × 10^11^ CFU/day, and was administered alongside standardized UC treatments, most notably mesalazine at 2.0–4.0 g/day. Table 2 summarizes the recent clinical data on the use of multi-strain probiotics for UC treatment.

### 5.1. Clinical Outcomes of Low-Complexity Multi-Strain Probiotics (2–4 Strains) in Ulcerative Colitis

The use of triple Bifid formulations (comprising *Bifidobacterium longum*, *Lactobacillus acidophilus*, and *Enterococcus faecalis*) demonstrated consistent clinical efficacy in randomized clinical trials across two independent cohorts of patients with mild to moderate UC treated with mesalazine [65,66]. Both studies reported high clinical response rates compared to placebo, ranging from 90.0% to 92.3%, along with significant improvement in disease activity scores. This clinical improvement was accompanied by robust modulation of the inflammatory response, characterized by reduced levels of pro-inflammatory cytokines, specifically IL-6, IL-8, and TNF-α, as well as upregulation of the anti-inflammatory cytokine IL-10 and reduced high-sensitivity C-reactive protein (hs-CRP). Furthermore, supplementation with this probiotic combination enhanced gut barrier integrity and systemic immunity, as demonstrated by reduced levels of intestinal permeability markers (D-lactic acid, endotoxin (ET), and diamine oxidase (DAO)), increased CD4+ counts, and a higher CD4/CD8 ratio. An observational study using a probiotic combination of *Lactobacillus acidophilus*, *Clostridium butyricum*, *Bacillus mesentericus,* and *Streptococcus faecalis* in a cohort with endoscopic remission but persistent bowel-related symptoms [72] showed significant improvement in stool frequency (3.3 ± 1.4 to 2.9 ± 1.3, *p* = 0.012) and Bristol stool scale scores (4.9 ± 0.9 to 4.3 ± 0.7, *p* < 0.001). This symptom relief contributed to an enhanced quality of life as assessed by the Short Inflammatory Bowel Disease Questionnaire (SIBDQ), with total scores increasing from 50.6 ± 8.3 to 53.6 ± 6.5 (*p* = 0.005) and significant improvement in bowel, systemic, and social domains (*p* < 0.05).

Conversely, two randomized clinical studies using a four-strain probiotic (Symprove^®^), a combination of *Lactobacillus rhamnosus*, *Lactobacillus plantarum*, *Lactobacillus acidophilus*, and *Enterococcus faecium*, yielded no significant findings. A study involving inactive UC patients with concurrent IBS symptoms found no significant improvement in symptoms in the probiotic-treated group (50.0%) compared with the placebo group (44.0%) [70]. A separate cohort of asymptomatic UC patients similarly showed no significant differences in total disease activity, with reductions observed in both the probiotic-treated group (4.4 ± 2.7 to 3.4 ± 3.1) and the placebo group (3.9 ± 2.7 to 2.8 ± 2.4, *p* = 0.39), and no improvement across all inflammatory bowel disease–quality of life (IBD-QOL) domains [71]. However, FC levels showed a downward trend in the probiotic-treated group with a mean reduction of −314 ± 719 (725 ± 726 to 411 ± 460), whereas levels increased in the placebo group (436 ± 451 to 626 ± 1057), although this remained statistically non-significant (*p* = 0.08).

### 5.2. Clinical Outcomes of High-Complexity Multi-Strain Probiotics (>5 Strains) in Ulcerative Colitis Patients

Mixed results were observed in three studies involving probiotic mixtures containing more than five strains in patients with mild to moderate UC. A randomized clinical trial by Agraib et al. (2022) [69] demonstrated significant benefits when a mixture of nine *Lactobacillus* and five *Bifidobacterium* species was supplemented in combination with mesalazine and/or azathioprine. The clinical efficacy showed a 100% clinical response in the probiotic-treated group, along with a significantly higher remission rate (66.7%) compared to the placebo group (41.7% and 25.0%, respectively). These clinical outcomes correlated with improvements in disease activity, as measured by the partial Mayo score (pMS), with a lower total score (1.33 ± 0.49 vs. 3.42 ± 1.78, *p* < 0.001), lower sub-score for stool frequency (0.00 ± 0.00 vs. 1.17 ± 1.19, *p* = 0.003), and lower global assessment score (0.42 ± 0.51 vs. 1.00 ± 0.74, *p* = 0.035) compared to the placebo group. Additionally, significant modulation of biochemical markers was observed, particularly increased IL-10 and hematological parameters (hemoglobin, hematocrit, and red blood cells), along with reductions in C-reactive protein (CRP) and IgA levels, highlighting the immunomodulatory effects of the probiotic mixture.

Similarly, an open-label, randomized trial using a mixture of six strains of *Lactobacillus plantarum*, *Lactobacillus acidophilus*, *Lactobacillus rhamnosus*, *Lactobacillus bifidus*, *Lactobacillus casei*, and *Bifidobacterium infantis* [68] reported a trend toward improvement in the Truelove and Witts disease score (52.9% vs. 23.5%, *p* = 0.07), and a significantly better histological index (82.3% vs. 41.1%, *p* = 0.03) compared to the control group. Specific symptom-based improvements observed in the probiotic-treated group included reductions in diarrhea (82.4% vs. 47.1%, *p* = 0.03) and blood in stool (70.6% vs. 17.6%, *p* = 0.002) relative to patients receiving mesalazine treatment only. In contrast, a randomized, placebo-controlled study supplemented with four *Lactobacillus* and four *Bifidobacterium* strains (Camflor^®^) [67] showed a significant improvement in the Lichtiger disease score within the probiotic-treated group, with a reduction from (4.3 ± 2.4 to 3.1± 1.4, *p* = 0.001). However, there was no significant improvement compared to the placebo group (3.1 ± 1.4 vs. 2.7 ± 1.6, *p* = 0.37). Moreover, the Mayo rectal bleeding score and FC level also showed no significant improvement within or between the groups (*p* > 0.05), despite slight reductions in both parameters across both study arms.

### 5.3. Clinical Application of Low-Complexity and High-Complexity Multi-Strain Probiotics in UC

Generally, the clinical application of probiotics has been positioned mainly as adjunctive therapy by major clinical guidelines, including the American Gastroenterological Association (AGA) and the European Crohn’s and Colitis Organization (ECCO). In particular, the AGA has restricted the use of probiotics solely to the context of clinical trials due to insufficient evidence, thereby limiting its recommendation for the induction or maintenance of remission in UC [74]. This caution arises from variability in the strains used, dosage, routes of administration, and study design, including differences in the reporting of endpoints and outcomes. The heterogeneity among studies assessing probiotics for UC treatment contributes to the uncertainty regarding the use of probiotics as a standardized treatment in UC.

Conversely, ECCO recognizes the use of specific, well-researched, evidence-based probiotic strains regarded as safe for human consumption, aimed at inducing and maintaining remission in mild to moderate disease [11]. VSL#3 is one of the high-complexity MSPs that has been consistently studied for its efficacy and safety in UC treatment [75,76,77,78]. Notably, a meta-analysis evaluating probiotic efficacy in IBD demonstrated that while probiotics as a broad category showed no significant benefit in inducing remission in active UC, sub-analyses of trials using VSL#3 demonstrated significant efficacy in inducing remission [79]. This finding highlights the clinical utility of high-complexity MSPs such as VSL#3 in UC management. However, the evidence for low-complexity MSPs is limited; therefore, their clinical application in UC management remains ambiguous.

## 6. Specificity vs. Synergy in Probiotic Therapy for UC Treatment

**Figure 4:**
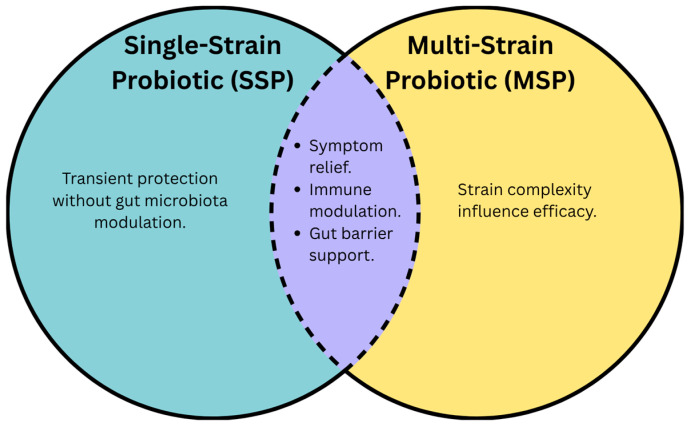
Beneficial role of single-strain probiotics (SSPs) and multi-strain probiotics (MSPs). Both approaches provide core benefits in symptomatic relief, immunomodulation, and gut barrier support. Specific differences were limitations in the modulation of gut microbiota by SSPs and the influence of strain complexity within MSPs, impacting the overall efficiency.

Current evidence suggests a potential role for both SSPs and MSPs in managing UC (Figure 4). Several included studies report that SSPs and MSPs are associated with improved clinical outcomes, including reduced disease activity and symptomatic relief, such as decreased diarrhea and bloody stools, although these findings were varied across studies. Probiotic supplementation has been shown to induce remission in patients with mild-to-moderate disease and maintain remission in patients with inactive disease [80]. This significant improvement in disease activity results from the effective control of systemic and intestinal inflammation, driven by the immunomodulatory properties of probiotics. Probiotics can modulate inflammatory cytokines by reducing the expression of IL-6, IL-8, and TNF-α, which are primarily associated with the T-helper 2/T-helper 17 (Th2/Th17) immune response, and by enhancing the production of the anti-inflammatory cytokine IL-10, thereby balancing the immune response within the gut microenvironment. Dysregulation of T-helper cells, particularly Th1, Th2, Th17, and Treg, significantly contributes to the degree of intestinal inflammation in UC patients [81,82], establishing its significance in clinical prediction of the disease [83]. Consequently, reductions in CRP and FC are indicative of a successful therapeutic response correlated with systemic and intestinal inflammation, both of which are essential non-invasive markers for monitoring disease severity and mucosal healing in UC [84,85,86].

When inflammation is normalized, the gut barrier undergoes structural and functional restoration. This is associated with a significant reduction in intestinal permeability markers (D-lactic acid, ET, and DAO levels). These markers are used to assess intestinal barrier damage and are often elevated in patients with intestinal disease [87]. A study by Zhang et al. (2022) [88] demonstrated that these markers correlate with predicting endoscopic remission, where a reduction in these markers indicates restoration of the ‘leaky gut’ in patients with UC. This reduction was also associated with improvements in endoscopic and histological indices among patients in the study cohort. Therefore, this highlights the role of probiotics in strengthening the gut barrier and supporting mucosal healing. Achieving endoscopic and histologic remission is now the new paradigm in the current treat-to-target approach for UC disease management, ultimately leading to deep remission and improved long-term outcomes [89]. Despite this evidence, the substantial methodological and clinical heterogeneity across included studies limits the certainty of these findings, which should therefore be interpreted as indicative rather than conclusive.

Despite their enhanced effects on UC treatment, some limitations are observed among probiotic types. In particular, the functionality of SSPs may be sufficient to improve clinical outcomes, yet they lack the capacity to fully restore microbial diversity. While SSPs are effective at inhibiting the growth of pathogenic species (Figure 1), they do not directly participate in rebuilding the complex gut microenvironment. In contrast, MSPs utilize cell-to-cell communication mechanisms that foster synergistic interactions between strains, as detailed in Figure 3, enabling them to occupy diverse ecological niches and provide complementary metabolites that promote the proliferation of beneficial species [42]. Once the gut microbiota stabilizes, it reaches a state of eubiosis, defined as an interspecies balance within the microbiota community [90]. An in vivo study involving healthy mice directly compared the effects of MSPs and SSPs on gut microbiota modulation, demonstrating that MSPs more effectively reshaped and diversified the microbial community structure following treatment [91].

On the other hand, strain complexity may influence clinical outcomes in MSPs. Studies with low-strain-count MSPs showed consistent findings compared to those using high-strain-count MSPs among patients with mild-to-moderate disease. These discrepancies may be due to differences in standard medication. In trials with high strain count MSPs, successful outcomes were observed when standardized treatments such as mesalazine or a combination of mesalazine and azathioprine were used. Conversely, unsuccessful trials often involved a diverse range of medications, including biologics. The inclusion of biologics may mask the true efficacy of probiotics, as these potent agents independently improve disease activity and inflammatory markers that overlap with the intended benefits of probiotics. Clinical trials involving patients with inactive disease observed minimal therapeutic improvements. This lack of significant change is likely attributable to a ‘ceiling effect,’ as patients already in remission exhibit normalized disease activity and baseline inflammatory markers. Despite the absence of statistically significant improvements, these cohorts consistently maintained remission following the intervention. This suggests that probiotics may play a role in remission maintenance, even if the improvements did not reach statistical significance.

## 7. Fecal Microbiota Transplantation (FMT) in Ulcerative Colitis Treatment

Besides probiotics, fecal microbiota transplantation (FMT) is another microbiome-based treatment for UC. FMT has become a promising microbiome-based therapeutic strategy for inducing clinical and endoscopic remission in patients with mild-to-moderate active UC [92]. Unlike its established use in recurrent *Clostridioides difficile* infection, the application of FMT in UC remains under active investigation and typically requires intensive, repeated administration protocols [93]. Clinical trials and meta-analyses have demonstrated that FMT is significantly more effective than placebo in achieving clinical remission and endoscopic improvement [94]. Response rates with FMT reach 94% in FMT-treated patients, compared with 70% in patients receiving conventional therapy [95].

The therapeutic effects of FMT are mediated through several interconnected mechanisms. First, FMT corrects intestinal dysbiosis by restoring microbial diversity and enriching beneficial SCFA-producing bacteria, particularly butyrate-producing species [96,97,98]. These microbial changes strengthen the intestinal barrier, promote epithelial regeneration, and reduce mucosal inflammation in UC [98]. In addition, FMT exerts potent anti-inflammatory effects by significantly reducing circulating inflammatory mediators, including CRP, TNF-α, and IL-6 [95]. The suppression of these pro-inflammatory cytokines contributes to the attenuation of chronic intestinal inflammation, which is essential in pathogenesis.

Despite microbial balance, FMT restores immune homeostasis. Studies have shown that FMT regulates peripheral T-cell subsets by increasing the proportions of CD3+ and CD4+ T lymphocytes while reducing excessive CD8+ T-cell activity, thereby improving the CD4+/CD8+ ratio [95,99,100]. Furthermore, FMT decreases elevated immunoglobulin levels, including IgA, IgG, and IgM, thereby reducing abnormal immune activation and limiting immune-mediated tissue injury [95,101].

Physiologically, FMT influences gastrointestinal motility by regulating gut hormones through microbiota-mediated mechanisms [102]. Dysbiosis-associated alterations in intestinal flora can disrupt normal gastrointestinal motility [102]. For instance, motilin levels are reduced to suppress excessive intestinal contractions, while concentrations of cholecystokinin and vasoactive intestinal peptide increase [95,103]. This promotes coordinated gastrointestinal transit and functional recovery. Through the combined effects of microbiota restoration, inflammation suppression, immune regulation, and regulating gastrointestinal motility, FMT provides a multifaceted therapeutic approach for the management of UC.

Current FMT protocols generally involve carefully selected adult or pediatric patients with mild-to-moderate UC and employ repeated dosing regimens delivered through colonoscopy, sigmoidoscopy, retention enemas, or encapsulated formulations [93,104]. To ensure consistency and maximize microbial engraftment, clinical studies commonly utilize frozen stool material obtained from a single screened donor throughout the treatment course [93]. Although FMT is generally well tolerated, mild adverse effects such as abdominal discomfort, bloating, diarrhea, and transient fever may occur [105]. Therefore, FMT is not currently considered a first-line therapy for UC and is primarily recommended within regulated clinical trial settings or specialized treatment programs [106].

## 8. Limitations and Future Directions

Despite the therapeutic potential of probiotic interventions, several limitations exist in clinical research. There is significant heterogeneity among the studies included in this narrative, which hinders direct comparison. The clinical application of probiotics is often complicated by host-related factors, such as age, sex, dietary intake, disease characteristics and severity, medication, IBD family history, and treatment-related factors, such as dosage, strain composition, and duration [107]. Moreover, differences in study design, particularly in methodological aspects, pose a meaningful challenge for drawing a definitive comparative conclusion regarding the relative clinical efficacy of SSPs versus MSPs.

In addition, the wide variety of clinical and molecular endpoints measured across studies makes it challenging to determine the exact effect of probiotic interventions. These endpoints range from subjective, index-based measures such as disease activity and quality-of-life assessments, to objective, index-based measures such as endoscopic and histological parameters, to biochemical markers for disease monitoring, and to molecular techniques, including gene and protein expression analysis and microbiome analysis. The absence of a standardized outcome measurement framework limits our ability to draw robust conclusions regarding the comparative efficacy of SSPs and MSPs.

Notably, there is a lack of head-to-head comparative studies directly evaluating the efficacy of SSPs versus MSPs in UC treatment. Although some systematic reviews have examined the comparative efficacy of probiotics across various disease conditions, most clinical studies evaluate single-strain or multi-strain formulations against a control or placebo rather than in direct comparison, thereby limiting the available evidence on their relative performance [108,109,110,111]. To address this limitation, we suggest that a future direct head-to-head clinical evaluation between SSPs and MSPs, using standardized outcome measures, would provide a more comprehensive understanding of their respective clinical outcomes and mechanisms of action in inducing and maintaining remission in patients with UC.

Current evidence also suggests that disease-specific variables, such as the distinction between active and inactive disease states, significantly influence microbial response [112]. Disease severity directly affects the composition of the gut microbiota. As the disease progresses toward a severe state, there is a gradual increase in pathogenic species and a reduction in beneficial species [113]. Therefore, modulation of the gut microbiota is particularly effective during mild-to-moderate disease by reducing inflammation and restoring gut microbiota balance as opposed to the inactive state, where the gut composition is relatively more stable.

Moreover, the categorization of MSPs into low- and high-complexity formulations in this review was based on a functional rationale relating to strain composition and inter-genus compatibility, which can lead to functional redundancy and antagonistic interactions. This could contribute to strain incompatibility, which is a critical determinant of probiotics’ efficacy, as strains within a formulation must work synergistically to convey their intended beneficial properties [114]. An increasing number of strains does not necessarily improve probiotic functionality, and a mixture of various strain species with different effects may provide greater efficacy [115,116]. Therefore, the consideration of applying this categorization is solely to observe the pattern of probiotics mixture in terms of strain variability. However, we acknowledge that this framework is not a universally validated biological or clinical cutoff. Therefore, future research could establish evidence-based criteria for defining probiotic complexity in clinical applications.

The field is now shifting toward personalized probiotic therapy designed to complement the unique clinical and microbial profiles of individual patients [117]. However, widespread application remains limited by high implementation costs and restricted accessibility [118]. Therefore, future research should prioritize expanding the use of personalized probiotics, with a specific focus on clinical studies to evaluate their safety and tolerability [119]. This is essential to provide a deeper understanding of the underlying mechanisms and therapeutic efficacy of these tailored interventions [120].

## 9. Conclusions

In conclusion, the clinical evidence presented for both SSPs and MSPs reported favorable findings in UC management, including improvement in clinical symptoms, reduction of inflammation, and restoration of gut barrier function. SSPs appear to exert a targeted, strain-specific effect, while MSPs may act through complementary synergistic mechanisms. However, the overall certainty of evidence remains limited by significant methodological and disease phenotype heterogeneity across included studies. Hence, a comparative conclusion regarding the relative efficacy of both approaches should be drawn with caution, pending further high-quality evidence. These limits underscore the need for a direct, head-to-head clinical trial using a standardized outcome framework to compare the mechanisms of action of both SSPs and MSPs. A future direction should shift from generalized probiotics to personalized probiotics to address the complexity of the disease and optimize clinical outcomes in UC.

## Figures and Tables

**Table 1:** Summary of selected clinical studies evaluating the efficacy of single-strain probiotics in ulcerative colitis.

Strain	Sample Size (*n*)	Duration (Weeks)	Daily Dosage	Clinical Data	Paraclinical Data	Study
EcN	49	8	2 caps (W1–W4), then 1 cap (W5–W8)	-	↓ FC post-EcN (*p* < 0.001)	[37]
	133	8	Escalating dose, 2.5 × 10^9^ CFU (day 1–4), then 5 × 10^9^ CFU from day 5.	↑ clinical response at 4 weeks (39.7% vs. 21.7%, *p* = 0.04), ↑ rate of IBDQ score reduction (13.3% vs. 1.7%, *p* = 0.02).	↔ microbial diversity, ↑ *E. coli* (EcN-treated group), ↑ Endoscopic remission after 8 weeks (46.4% vs. 27.1%; *p* = 0.03)	[39]
	94	52	5 × 10^9^ CFU	↓ pMS (*p* = 0.025).	↔ FC at 3 and 6 months (*p* > 0.05).	[38]
LGG	75	4	SD: 1.2 × 10^10^ CFU DD: 2.4 × 10^10^ CFU	Total response 58%, stable 38%, worsened 4%, ↓ in pMS for both SD and DD (*p* < 0.05)	↓ endoscopic (subset (*n* = 27), *p* < 0.05), ↔ efficacy and safety between doses (*p* > 0.05)	[40]

FC, fecal calprotectin; IBDQ, inflammatory bowel disease questionnaire; pMS, partial Mayo score; CFU, colony-forming unit; EcN, *Escherichia coli* Nissle 1917; LGG, *Lactobacillus rhamnosus* GG; SD, standard dose; DD, double dose; W, week; cap/caps, capsule(s); UC, ulcerative colitis; ↑, increase/improve; ↓, decrease/reduction; ↔, no significant changes.

**Table 2:** Summary of the selected clinical studies evaluating the efficacy of multi-strain probiotics in ulcerative colitis.

Strain Count	Brand/ Strain Composition	Sample Size (*n*)	Duration (Weeks)	Daily Dosage	Clinical Data	Paraclinical Data	Study
2–4 strains	Bifid triple formulation (*Bifidobacterium longum*, *Lactobacillus acidophilus*, *Enterococcus faecalis*)	130	8	420 mg/cap, 1 cap × 3 times	↑ clinical response (92.3% vs. 76.9%, *p* = 0.015)	↓ inflammation level (IL-6, IL-8, hs-CRP, TNF-α) and intestinal permeability marker (D-lactic acid, ET, DAO) (*p* < 0.05), ↑ Immunity levels (CD4+, CD4/CD8) (*p* < 0.05)	[65]
		120	8	3 × 10^6^ CFU, 2 cap × 3 times	↑ clinical response (90.0% vs. 73.3%, *p* < 0.05), ↓ UCDAI score (*p* < 0.05)	↓ TNF-α and IL-8; ↑ IL-10 (*p* < 0.05)	[66]
	Symprove^®^ (*Lactobacillus rhamnosus* NCIMB 30174, *Lactobacillus plantarum* NCIMB 30173, *Lactobacillus acidophilus* NCIMB 30175, *Enterococcus faecium* NCIMB 30176)	61	12	2.5 × 10^10^ CFU/day	↔ IBS symptoms (50.0% vs. 44.0%, *p* = 1.00)		[70]
		81	4	1 × 10^10^ CFU/50 mL, 1 mL/kg/day	↔ IBD-QOL domains (*p* > 0.05), ↔ total disease activity (*p* = 0.39)	↔ FC levels (*p* = 0.08), a downward trend was observed in the probiotic-treated group (mean reduction, −314 ± 719)	[71]
	Biotop^®^ (*Lactobacillus acidophilus*, *Clostridium butyricum* TO-A, *Bacillus mesentericus* TO-A, *Streptococcus faecalis* T-110)	43	4	75 mg (*L. acidophilus*), 25 mg (*C. butyricum*), 25 mg (*B. mesentericus*), 5 mg (*S. faecalis*)/cap × 3 times	↑ total SIBDQ (50.6 to 53.6, *p* = 0.005), domain specific (bowel, systemic, and social) (*p* < 0.05), Improvement in stool frequency and Bristol stool score (*p* < 0.05)	-	[72]
>5 strains	*Lactobacillus acidophilus*, *Lactobacillus bulgaricus*, *Lactobacillus plantarum*, *Bifidobacterium longum*, *Bifidobacterium breve*, *Bifidobacterium infantis*, *Streptococcus thermophilus*	60	16	9 × 10^11^ CFU/day	Improvement in Lichtiger score within group (*p* = 0.001), no differences against placebo (*p* = 0.37)	↔ FC levels and Mayo rectal bleeding score within and between groups (*p* > 0.05)	[67]
	*Lactobacillus plantarum*, *Lactobacillus acidophilus*, *Lactobacillus rhamnosus*, *Lactobacillus bifidus*, *Lactobacillus casei*, *Bifidobacterium infantis*	17	12	4 × 10^7^ CFU/day	↑ disease activity index (52.9% vs. 23.5%, *p* = 0.07), ↓ specific symptoms: diarrhea (82.4% vs. 47.1%, *p* = 0.03) and blood in stool (70.6% vs. 17.6%, *p* = 0.002)	Histologic index (82.3% vs. 41.1%, *p* = 0.03)	[68]
	*Lactobacillus paracasei* (A234), *Lactobacillus gasseri* (A237), *Lactobacillus rhamnosus* (A119, A193), *Lactobacillus acidophilus* (A118), *Lactobacillus plantarum* (A138), *Lactobacillus casei* (A179), *Lactobacillus reuteri* (A113), *Lactococcus lactis* (A328), *Bifidobacterium animalis* subsp. *lactis* (A026), *Bifidobacterium breve* (A055), *Bifidobacterium longum* subsp. *longum* (A027), *Bifidobacterium bifidum* (A058), *Bifidobacterium longum* subsp. *infantis* (A041)	30	6	3 × 10^10^ CFU/day	↑ remission rate (66.7% vs. 25.0%, *p* = 0.003), ↑ clinical response (100% vs. 41.7%, *p* = 0.002), ↓ stool frequency, global assessment, and total pMS score in the probiotic group (*p* < 0.05)	↑ IL-10, hemoglobin, hematocrit, and red blood cell levels (*p* < 0.05), ↓ CRP level, IgA level (*p* < 0.05)	[69]

CFU, colony-forming unit; mg, milligrams; mL, milliliters; kg, kilograms; cap/caps, capsule(s); IL, interleukin; TNF-α, tumor necrosis factor-alpha; hs-CRP/CRP, (high sensitivity) C-reactive protein; ET, endotoxin; DAO, diamine oxidase; CD, cluster of differentiation; FC, fecal calprotectin; IgA, Immunoglobulin A; pMS, partial Mayo score; UCDAI, ulcerative colitis disease activity index; IBD-QOL, inflammatory bowel disease–quality of life; SIBDQ, Short Inflammatory Bowel Disease Questionnaire; UC, ulcerative colitis; IBS, irritable bowel syndrome; ↑, increase/improve; ↓, decrease/reduction; ↔, no significant changes.

## Data Availability

No new data were created or analyzed in this study. Data sharing is not applicable to this article.

## Abbreviations

The following abbreviations are used in this manuscript:

UC	Ulcerative colitis
SSPs	Single-strain probiotics
MSPs	Multi-strain probiotics
IBD	Inflammatory bowel disease
5-ASAs	5-aminosalicylates
RCT	Randomized clinical trial
EcN	*Escherichia coli* strain Nissle 1917
LGG	*Lactobacillus rhamnosus* GG
Nrf2	Nuclear factor erythroid 2-related factor 2
IgA	Immunoglobulin A
IL-2	Interleukin-2
IL-4	Interleukin-4
IL-12	Interleukin-12
TNF-α	Tumor necrosis factor-alpha
IFN-γ	Interferon-gamma
IL-10	Interleukin-10
TGF-β	Transforming growth factor-beta
ZO-1	Zonula Occludens-1
ZO-2	Zonula Occludens-2
CFU	Colony-forming unit
DB	Double blind
PC	Placebo
OL	Open-label
DC	Dose comparison
RS	Retrospective study
RC	Retrospective control
FC	Fecal calprotectin
IBDQ	Inflammatory bowel disease questionnaire
pMS	Partial Mayo score
SD	Standard dose
DD	Double dose
g	Gram
pABA	Para-aminobenzoic acid
SCFAs	Short-chain fatty acids
GVB	Gut vascular barrier
PV1	Plasmalemma vesicle-associated protein-1
NFκB	Nuclear factor-kappa-B
IL-6	Interleukin-6
IL-8	Interleukin-8
NKCs	Natural killer cells
Th1	T-helper 1 cells
CD	Cluster of differentiation
IBS	Irritable bowel syndrome
OB	Observational study
mg	Milligram
kg	Kilogram
Cap/caps	Capsule
Hs-CRP	High-sensitivity C-reactive protein
CRP	C-reactive protein
UCDAI	Ulcerative Colitis Disease Activity Index
IBD-QOL	Inflammatory bowel disease–quality of life
SIBDQ	Short Inflammatory Bowel Disease Questionnaire
ET	Endotoxin
DAO	Diamine oxidase
Th2	T-helper 2 cells
Th17	T-helper 17 cells
Treg	T-regulatory cells
AIPs	Auto-inducing proteins
IECs	Intestinal epithelial cells

## References

[1] Le Berre C., Honap S., Peyrin-Biroulet L. (2023). Ulcerative colitis. Lancet.

[2] Hracs L., Windsor J.W., Gorospe J., Cummings M., Coward S., Buie M.J., Quan J., Goddard Q., Caplan L., Markovinović A. (2025). Global evolution of inflammatory bowel disease across epidemiologic stages. Nature.

[3] Landeira M., Markert M., Guedes S., Balijepalli C., Druyts E., Nielsen L. (2026). P1186 Global epidemiology of Ulcerative Colitis: A systematic literature review from 2014–2025. J. Crohn’s Colitis.

[4] Wang R., Li Z., Liu S., Zhang D. (2023). Global, regional and national burden of inflammatory bowel disease in 204 countries and territories from 1990 to 2019: A systematic analysis based on the Global Burden of Disease Study 2019. BMJ Open.

[5] Roberts-Thomson I.C., Bryant R.V., Costello S.P. (2019). Uncovering the cause of ulcerative colitis. JGH Open.

[6] Pandey H., Jain D., Tang D.W.T., Wong S.H., Lal D. (2024). Gut microbiota in pathophysiology, diagnosis, and therapeutics of inflammatory bowel disease. Intest. Res..

[7] Shen Y., Fan N., Ma S.X., Cheng X., Yang X., Wang G. (2025). Gut Microbiota Dysbiosis: Pathogenesis, Diseases, Prevention, and Therapy. MedComm.

[8] Di Vincenzo F., Del Gaudio A., Petito V., Lopetuso L.R., Scaldaferri F. (2024). Gut microbiota, intestinal permeability, and systemic inflammation: A narrative review. Intern. Emerg. Med..

[9] Macura B., Kiecka A., Szczepanik M. (2024). Intestinal permeability disturbances: Causes, diseases and therapy. Clin. Exp. Med..

[10] Swaminathan A., Day A.S., Sparrow M.P., Peyrin-Biroulet L., Siegel C.A., Gearry R.B. (2024). Measuring disease severity in inflammatory bowel disease—Beyond treat to target. Aliment. Pharmacol. Ther..

[11] Raine T., Bonovas S., Burisch J., Kucharzik T., Adamina M., Annese V., Bachmann O., Bettenworth D., Chaparro M., Czuber-Dochan W. (2022). ECCO Guidelines on Therapeutics in Ulcerative Colitis: Medical Treatment. J. Crohn’s Colitis.

[12] Alshehri D., Saadah O., Mosli M., Edris S., Alhindi R., Bahieldin A. (2021). Dysbiosis of gut microbiota in inflammatory bowel disease: Current therapies and potential for microbiota-modulating therapeutic approaches. Biomol. Biomed..

[13] Hill C., Guarner F., Reid G., Gibson G.R., Merenstein D.J., Pot B., Morelli L., Canani R.B., Flint H.J., Salminen S. (2014). The International Scientific Association for Probiotics and Prebiotics consensus statement on the scope and appropriate use of the term probiotic. Nat. Rev. Gastroenterol. Hepatol..

[14] Plaza-Diaz J., Ruiz-Ojeda F.J., Gil-Campos M., Gil A. (2019). Mechanisms of Action of Probiotics. Adv. Nutr..

[15] Mazziotta C., Tognon M., Martini F., Torreggiani E., Rotondo J.C. (2023). Probiotics Mechanism of Action on Immune Cells and Beneficial Effects on Human Health. Cells.

[16] Štofilová J., Kvaková M., Kamlárová A., Hijová E., Bertková I., Guľašová Z. (2022). Probiotic-Based Intervention in the Treatment of Ulcerative Colitis: Conventional and New Approaches. Biomedicines.

[17] Ouwehand A.C. (2017). A review of dose-responses of probiotics in human studies. Benef. Microbes.

[18] Morelli L., Pellegrino P. (2021). A critical evaluation of the factors affecting the survival and persistence of beneficial bacteria in healthy adults. Benef. Microbes.

[19] McFarland L.V. (2021). Efficacy of Single-Strain Probiotics Versus Multi-Strain Mixtures: Systematic Review of Strain and Disease Specificity. Dig. Dis. Sci..

[20] Kesavelu D., Yadav Krishnamurty A., A P. (2025). Single Strain vs. Multiple Strain Probiotics: The Clinician’s Choice. Cureus.

[21] Chapman C.M.C., Gibson G.R., Rowland I. (2011). Health benefits of probiotics: Are mixtures more effective than single strains?. Eur. J. Nutr..

[22] Pisani A., Rausch P., Bang C., Ellul S., Tabone T., Marantidis Cordina C., Zahra G., Franke A., Ellul P. (2022). Dysbiosis in the Gut Microbiota in Patients with Inflammatory Bowel Disease during Remission. Microbiol. Spectr..

[23] Macfarlane S., Furrie E., Kennedy A., Cummings J.H., Macfarlane G.T. (2005). Mucosal bacteria in ulcerative colitis. Br. J. Nutr..

[24] Han T., Zhang Y., Zheng G., Guo Y. (2025). From pathogenic mechanisms to therapeutic perspectives: A review of gut microbiota and intestinal mucosal immunity in inflammatory bowel disease. Front. Immunol..

[25] Furrie E., Macfarlane S., Cummings J.H., Macfarlane G.T. (2004). Systemic antibodies towards mucosal bacteria in ulcerative colitis and Crohn’s disease differentially activate the innate immune response. Gut.

[26] Bu S., Cheng X., Chen M., Yu Y. (2025). Ulcerative Colitis: Advances in Pathogenesis, Biomarkers, and Therapeutic Strategies. Pharmacogenomics Pers. Med..

[27] Macfarlane S., Furrie E., Cummings J.H., Macfarlane G.T. (2004). Chemotaxonomic Analysis of Bacterial Populations Colonizing the Rectal Mucosa in Patients with Ulcerative Colitis. Clin. Infect. Dis..

[28] Parada Venegas D., De La Fuente M.K., Landskron G., González M.J., Quera R., Dijkstra G., Harmsen H.J.M., Faber K.N., Hermoso M.A. (2019). Short Chain Fatty Acids (SCFAs)-Mediated Gut Epithelial and Immune Regulation and Its Relevance for Inflammatory Bowel Diseases. Front. Immunol..

[29] Muro P., Zhang L., Li S., Zhao Z., Jin T., Mao F., Mao Z. (2024). The emerging role of oxidative stress in inflammatory bowel disease. Front. Endocrinol..

[30] Pastorelli L., De Salvo C., Mercado J.R., Vecchi M., Pizarro T.T. (2013). Central Role of the Gut Epithelial Barrier in the Pathogenesis of Chronic Intestinal Inflammation: Lessons Learned from Animal Models and Human Genetics. Front. Immunol..

[31] Scaldaferri F., Gerardi V., Mangiola F., Lopetuso L.R., Pizzoferrato M., Petito V., Papa A., Stojanovic J., Poscia A., Cammarota G. (2016). Role and mechanisms of action of *Escherichia coli* Nissle 1917 in the maintenance of remission in ulcerative colitis patients: An update. World J. Gastroenterol..

[32] Güttsches A.-K., Löseke S., Zähringer U., Sonnenborn U., Enders C., Gatermann S., Bufe A. (2012). Anti-inflammatory modulation of immune response by probiotic *Escherichia coli* Nissle 1917 in human blood mononuclear cells. Innate Immun..

[33] Bartram E., Asai M., Gabant P., Wigneshweraraj S. (2023). Enhancing the antibacterial function of probiotic *Escherichia coli* Nissle: When less is more. Appl. Environ. Microbiol..

[34] Leser T., Baker A. (2024). Molecular Mechanisms of *Lacticaseibacillus rhamnosus*, LGG^®^ Probiotic Function. Microorganisms.

[35] Si W., Zhao X., Li R., Li Y., Ma C., Zhao X., Bugno J., Qin Y., Zhang J., Liu H. (2025). *Lactobacillus rhamnosus* GG induces STING-dependent IL-10 in intestinal monocytes and alleviates inflammatory colitis in mice. J. Clin. Investig..

[36] Hu R., Yang T., Ai Q., Shi Y., Ji Y., Sun Q., Tong B., Chen J., Wang Z. (2024). Autoinducer-2 promotes the colonization of *Lactobacillus rhamnosus* GG to improve the intestinal barrier function in a neonatal mouse model of antibiotic-induced intestinal dysbiosis. J. Transl. Med..

[37] Bodini G., Ghezzi A., Pasta A., Marabotto E., Calabrese F., Facchini C., Demarzo M.G., Giannini E.G. (2023). Reduction of Fecal Calprotectin Levels Induced by a Short Course of *Escherichia Coli* Nissle is Associated with a Lower Likelihood of Disease Flares in Patients with Ulcerative Colitis in Clinical Remission. J. Gastrointest. Liver Dis..

[38] Oh G.M., Moon W., Seo K.I., Jung K., Kim J.H., Kim S.E., Park M.I., Park S.J. (2021). Therapeutic Potential of *Escherichia coli* Nissle 1917 in Clinically Remission-attained Ulcerative Colitis Patients: A Hospital-based Cohort Study. Korean J. Gastroenterol..

[39] Park S.-K., Kang S.-B., Kim S., Kim T.O., Cha J.M., Im J.P., Choi C.H., Kim E.S., Seo G.S., Eun C.S. (2022). Additive effect of probiotics (Mutaflor) on 5-aminosalicylic acid therapy in patients with ulcerative colitis. Korean J. Intern. Med..

[40] Pagnini C., Di Paolo M.C., Urgesi R., Pallotta L., Fanello G., Graziani M.G., Delle Fave G. (2023). Safety and Potential Role of *Lactobacillus rhamnosus* GG Administration as Monotherapy in Ulcerative Colitis Patients with Mild–Moderate Clinical Activity. Microorganisms.

[41] Kaur K. (2025). Application and Challenges of Using Probiotic Lactobacillus and Bifidobacterium to Enhance Overall Health and Manage Diseases. Diseases.

[42] Kwoji I.D., Aiyegoro O.A., Okpeku M., Adeleke M.A. (2021). Multi-Strain Probiotics: Synergy among Isolates Enhances Biological Activities. Biology.

[43] Davares A.K.L., Arsene M.M.J., Viktorovna P.I., Vyacheslavovna Y.N., Vladimirovna Z.A., Aleksandrovna V.E., Nikolayevich S.A., Nadezhda S., Anatolievna G.O., Nikolaevna S.I. (2022). Quorum-Sensing Inhibitors from Probiotics as a Strategy to Combat Bacterial Cell-to-Cell Communication Involved in Food Spoilage and Food Safety. Fermentation.

[44] Naoun A.A., Raphael I., Forsthuber T.G. (2022). Immunoregulation via Cell Density and Quorum Sensing-like Mechanisms: An Underexplored Emerging Field with Potential Translational Implications. Cells.

[45] Salman M.K., Abuqwider J., Mauriello G. (2023). Anti-Quorum Sensing Activity of Probiotics: The Mechanism and Role in Food and Gut Health. Microorganisms.

[46] Hmar E.B.L., Paul S., Sharma H.K. (2024). An Insight into the Combination of Probiotics and their Implications for Human Health. Endocr. Metab. Immune Disord. Drug Targets.

[47] Turroni F., Özcan E., Milani C., Mancabelli L., Viappiani A., Van Sinderen D., Sela D.A., Ventura M. (2015). Glycan cross-feeding activities between bifidobacteria under in vitro conditions. Front. Microbiol..

[48] Leblanc J.G., Milani C., De Giori G.S., Sesma F., Van Sinderen D., Ventura M. (2013). Bacteria as vitamin suppliers to their host: A gut microbiota perspective. Curr. Opin. Biotechnol..

[49] Yan D., Ye S., He Y., Wang S., Xiao Y., Xiang X., Deng M., Luo W., Chen X., Wang X. (2023). Fatty acids and lipid mediators in inflammatory bowel disease: From mechanism to treatment. Front. Immunol..

[50] Zhang X., Wu Y., Jiang Y., Fan J., Dong Y., Fang S., Zhu J., Gu S. (2025). High-potency multi-strain probiotic formulations for safety and improvement of gastrointestinal function and intestinal health: A randomized controlled clinical trial. Front. Nutr..

[51] Di Vito R., Conte C., Traina G. (2022). A Multi-Strain Probiotic Formulation Improves Intestinal Barrier Function by the Modulation of Tight and Adherent Junction Proteins. Cells.

[52] Naso A.M., Lizier M., Correale C., Silvestri A., Penna G., Brescia P., Rescigno M. (2025). A multi-strain probiotic formulation preserves intestinal epithelial and vascular barriers during enteropathogenic infection. Front. Microbiol..

[53] Alshihmani A.H.H., Kolahdooz H., Mahmoudi M., Rezaieyazdi Z., Kamal Kheder R., Tabasi N.S., Fadaee A., Esmaeili S.A. (2025). Immunomodulatory Effects of Multi-Strain Probiotic Capsules for Psoriatic Arthritis: A Pilot Double-Blind Randomized Controlled Trial. Food Sci. Nutr..

[54] Niu C., Wang J., Lu X., Yu Y. (2026). Probiotics for ulcerative colitis: Mechanisms, therapeutic advances, and emerging strategies. Front. Microbiol..

[55] Wójcik R., Małaczewska J., Tobolski D., Miciński J., Kaczorek-Łukowska E., Zwierzchowski G. (2024). The Effect of Orally Administered Multi-Strain Probiotic Formulation (*Lactobacillus*, *Bifidobacterium*) on the Phagocytic Activity and Oxidative Metabolism of Peripheral Blood Granulocytes and Monocytes in Lambs. Int. J. Mol. Sci..

[56] Aziz N., Bonavida B. (2016). Activation of Natural Killer Cells by Probiotics. Forum Immunopathol. Dis. Ther..

[57] Kasti A.N., Synodinou K.D., Pyrousis I.A., Nikolaki M.D., Triantafyllou K.D. (2021). Probiotics Regulating Inflammation via NLRP3 Inflammasome Modulation: A Potential Therapeutic Approach for COVID-19. Microorganisms.

[58] Zhao L., Huang Y., Ye Z., Chen W., Zhang N., Wen Z., Ge C. (2026). Short-chain fatty acids attenuate sepsis-induced gut dysbiosis and hippocampal neuroinflammation via NLRP6 inflammasome activation in mice. Int. J. Surg..

[59] Zhang H., Zhao T., Gu J., Tang F., Zhu L. (2025). Gut microbiota and inflammasome-mediated pyroptosis: A bibliometric analysis from 2014 to 2023. Front. Microbiol..

[60] Chen Y., Ye X., Escames G., Lei W., Zhang X., Li M., Jing T., Yao Y., Qiu Z., Wang Z. (2023). The NLRP3 inflammasome: Contributions to inflammation-related diseases. Cell. Mol. Biol. Lett..

[61] Alkushi A.G., Elazab S.T., Abdelfattah-Hassan A., Mahfouz H., Salem G.A., Sheraiba N.I., Mohamed E.A.A., Attia M.S., El-Shetry E.S., Saleh A.A. (2022). Multi-Strain-Probiotic-Loaded Nanoparticles Reduced Colon Inflammation and Orchestrated the Expressions of Tight Junction, NLRP3 Inflammasome and Caspase-1 Genes in DSS-Induced Colitis Model. Pharmaceutics.

[62] Yuan J., Zhao F., Liu Y., Liu H., Zhang K., Tian X., Mu Y., Zhao J., Wang Y. (2023). Effects of Lactiplantibacillus plantarum on oxidative stress, mitophagy, and NLRP3 inflammasome activation in broiler breast meat. Poult. Sci..

[63] Zhao S., Chen F., Yin Q., Wang D., Han W., Zhang Y. (2020). Reactive Oxygen Species Interact with NLRP3 Inflammasomes and Are Involved in the Inflammation of Sepsis: From Mechanism to Treatment of Progression. Front. Physiol..

[64] Wang X., Zhang Q., Wang W., Wang X., Song B., Li J., Cui W., Jiang Y., Xie W., Tang L. (2025). Multi-Strain Probiotic Regulates the Intestinal Mucosal Immunity and Enhances the Protection of Piglets Against Porcine Epidemic Diarrhea Virus Challenge. Microorganisms.

[65] Li S., Yin Y., Xiao D., Zou Y. (2021). Supplemental bifid triple viable capsule treatment improves inflammatory response and T cell frequency in ulcerative colitis patients. BMC Gastroenterol..

[66] Huang M., Chen Z., Lang C., Chen J., Yang B., Xue L., Zhang Y. (2018). Efficacy of mesalazine in combination with bifid triple viable capsules on ulcerative colitis and the resultant effect on the inflammatory factors. Pak. J. Pharm. Sci..

[67] Tamizifar B., Feizi A., Rahim Khorasani M., Kassaian N., Zamanimoghadam A., Arbabnia S., Adibi Sede P. (2023). The effects of probiotics in ulcerative colitis patients: A randomized controlled double blind clinical trial. Funct. Foods Health Dis..

[68] Sánchez-Morales A., Pérez-Ayala M.F., Cruz-Martínez M., Arenas-Osuna J., Ramírez-Mendoza P., Ceniceros R.A., Mora-Cañas E.M., Cruz-Domínguez P., Saavedra-Salinas M. (2019). Probiotics’ effectiveness on symptoms, histological features and feeding tolerance in ulcerative colitis. Rev. Médica Inst. Mex. Seguro Soc..

[69] Agraib L.M., Yamani M.I., Tayyem R., Abu-Sneineh A.T., Rayyan Y.M. (2022). Probiotic supplementation induces remission and changes in the immunoglobulins and inflammatory response in active ulcerative colitis patients: A pilot, randomized, double-blind, placebo-controlled study. Clin. Nutr. ESPEN.

[70] Fennessy A., Doyle M., Boland A., Bourke R., O’Connor A. (2025). Four-strain probiotic exerts a positive effect on irritable bowel syndrome symptoms occurring in inflammatory bowel diseases in absence of inflammation (TRAIN-IBD trial). World J. Gastrointest. Pharmacol. Ther..

[71] Bjarnason I., Sission G., Hayee B.H. (2019). A randomised, double-blind, placebo-controlled trial of a multi-strain probiotic in patients with asymptomatic ulcerative colitis and Crohn’s disease. Inflammopharmacology.

[72] Lee J., Park S.B., Kim H.W., Lee H.S., Jee S.R., Lee J.H., Kim T.O. (2022). Clinical Efficacy of Probiotic Therapy on Bowel-Related Symptoms in Patients with Ulcerative Colitis during Endoscopic Remission: An Observational Study. Gastroenterol. Res. Pract..

[73] Jiang Y., Che L., Li S.C. (2025). Deciphering the personalized functional redundancy hierarchy in the gut microbiome. Microbiome.

[74] Su G.L., Ko C.W., Bercik P., Falck-Ytter Y., Sultan S., Weizman A.V., Morgan R.L. (2020). AGA Clinical Practice Guidelines on the Role of Probiotics in the Management of Gastrointestinal Disorders. Gastroenterology.

[75] Sood A., Midha V., Makharia G.K., Ahuja V., Singal D., Goswami P., Tandon R.K. (2009). The Probiotic Preparation, VSL#3 Induces Remission in Patients with Mild-to-Moderately Active Ulcerative Colitis. Clin. Gastroenterol. Hepatol..

[76] Miele E., Pascarella F., Giannetti E., Quaglietta L., Baldassano R.N., Staiano A. (2009). Effect of a Probiotic Preparation (VSL#3) on Induction and Maintenance of Remission in Children with Ulcerative Colitis. Am. J. Gastroenterol..

[77] Turcotte J.-F., Huynh H.Q. (2011). Treatment with the probiotic VSL#3 as an adjunctive therapy in relapsing mild-to-moderate ulcerative colitis significantly reduces ulcerative colitis disease activity. Evid. Based Med..

[78] Tursi A., Brandimarte G., Papa A., Giglio A., Elisei W., Giorgetti G.M., Forti G., Morini S., Hassan C., Pistoia M.A. (2010). Treatment of Relapsing Mild-to-Moderate Ulcerative Colitis with the Probiotic VSL#3 as Adjunctive to a Standard Pharmaceutical Treatment: A Double-Blind, Randomized, Placebo-Controlled Study. Am. J. Gastroenterol..

[79] Derwa Y., Gracie D.J., Hamlin P.J., Ford A.C. (2017). Systematic review with meta-analysis: The efficacy of probiotics in inflammatory bowel disease. Aliment. Pharmacol. Ther..

[80] Jadhav A., Jagtap S., Vyavahare S., Sharbidre A., Kunchiraman B. (2023). Reviewing the potential of probiotics, prebiotics and synbiotics: Advancements in treatment of ulcerative colitis. Front. Cell. Infect. Microbiol..

[81] Zhang Y., Shi W., Cao G., Li J., Wang H., Hao C. (2023). The significance of Th1,Th2,Th17and treg cells in the prediction and evaluation of ulcerative colitis. Eur. J. Inflamm..

[82] Gui X., Li J., Ueno A., Iacucci M., Qian J., Ghosh S. (2018). Histopathological Features of Inflammatory Bowel Disease are Associated with Different CD4+ T Cell Subsets in Colonic Mucosal Lamina Propria. J. Crohn’s Colitis.

[83] Fukaura K., Iboshi Y., Ogino H., Ihara E., Nakamura K., Nishihara Y., Nishioka K., Chinen T., Iwasa T., Aso A. (2019). Mucosal Profiles of Immune Molecules Related to T Helper and Regulatory T Cells Predict Future Relapse in Patients with Quiescent Ulcerative Colitis. Inflamm. Bowel Dis..

[84] Singh S., Ananthakrishnan A.N., Nguyen N.H., Cohen B.L., Velayos F.S., Weiss J.M., Sultan S., Siddique S.M., Adler J., Chachu K.A. (2023). AGA Clinical Practice Guideline on the Role of Biomarkers for the Management of Ulcerative Colitis. Gastroenterology.

[85] Steinsbø Ø., Aasprong O.G., Aabakken L., Karlsen L.N., Grimstad T. (2025). Fecal Calprotectin Correlates with Disease Extent but Remains a Reliable Marker of Mucosal Healing in Ulcerative Colitis. Am. J. Gastroenterol..

[86] Croft A., Lord A., Radford-Smith G. (2022). Markers of Systemic Inflammation in Acute Attacks of Ulcerative Colitis: What Level of C-reactive Protein Constitutes Severe Colitis?. J. Crohn’s Colitis.

[87] Linsalata M., Riezzo G., Clemente C., D’Attoma B., Russo F. (2020). Noninvasive Biomarkers of Gut Barrier Function in Patients Suffering from Diarrhea Predominant-IBS: An Update. Dis. Markers.

[88] Zhang Q., Gao X., Wu J., Chen M. (2022). The Correlation between Endotoxin, D-Lactate, and Diamine Oxidase with Endoscopic Activity in Inflammatory Bowel Disease. Dis. Markers.

[89] Peyrin-Biroulet L., Sandborn W., Sands B.E., Reinisch W., Bemelman W., Bryant R.V., D’Haens G., Dotan I., Dubinsky M., Feagan B. (2015). Selecting Therapeutic Targets in Inflammatory Bowel Disease (STRIDE): Determining Therapeutic Goals for Treat-to-Target. Am. J. Gastroenterol..

[90] Al-Rashidi H.E. (2022). Gut microbiota and immunity relevance in eubiosis and dysbiosis. Saudi J. Biol. Sci..

[91] He Q., Huang J., Zheng T., Lin D., Zhang H., Li J., Sun Z. (2021). Treatment with mixed probiotics induced, enhanced and diversified modulation of the gut microbiome of healthy rats. FEMS Microbiol. Ecol..

[92] Liu H., Li J., Yuan J., Huang J., Xu Y. (2023). Fecal microbiota transplantation as a therapy for treating ulcerative colitis: An overview of systematic reviews. BMC Microbiol..

[93] Lopetuso L.R., Deleu S., Puca P., Abreu M.T., Armuzzi A., Barbara G., Caprioli F., Chieng S., Costello S.P., Damiani A. (2025). Guidance for Fecal Microbiota Transplantation Trials in Ulcerative Colitis: The Second ROME Consensus Conference. Inflamm. Bowel Dis..

[94] Fang H., Fu L., Wang J. (2018). Protocol for Fecal Microbiota Transplantation in Inflammatory Bowel Disease: A Systematic Review and Meta-Analysis. BioMed Res. Int..

[95] Yang M., Li X., Zuo W., Gou J., Yu S., Huang M., Liu H. (2021). Efficacy of faecal microbiota transplantation on ulcerative colitis and its effect on gastrointestinal motility and immune function. Am. J. Transl. Res..

[96] Adamkova P., Hradicka P., Gancarcikova S., Kassayova M., Ambro L., Bertkova I., Maronek M., Farkasova Iannaccone S., Demeckova V. (2021). Single Donor FMT Reverses Microbial/Immune Dysbiosis and Induces Clinical Remission in a Rat Model of Acute Colitis. Pathogens.

[97] Hou S., Yu J., Li Y., Zhao D., Zhang Z. (2025). Advances in Fecal Microbiota Transplantation for Gut Dysbiosis-Related Diseases. Adv. Sci..

[98] Hamamah S., Gheorghita R., Lobiuc A., Sirbu I.-O., Covasa M. (2022). Fecal microbiota transplantation in non-communicable diseases: Recent advances and protocols. Front. Med..

[99] Wen X., Wang H.-G., Zhang M.-N., Zhang M.-H., Wang H., Yang X.-Z. (2021). Fecal microbiota transplantation ameliorates experimental colitis via gut microbiota and T-cell modulation. World J. Gastroenterol..

[100] Funderburg N., Stubblefield S., Sung H., Hardy G., Clagett B., Ignatz-Hoover J., Harding C., Fu P., Katz J., Lederman M. (2013). Circulating CD4^+^ and CD8^+^ T cells are activated in IBD and are associated with plasma markers of inflammation. Immunology.

[101] Huang W.-Q., Huang H.-L., Peng W., Liu Y.-D., Zhou Y.-L., Xu H.-M., Zhang L.-J., Zhao C., Nie Y.-Q. (2022). Altered Pattern of Immunoglobulin A-Targeted Microbiota in Inflammatory Bowel Disease After Fecal Transplantation. Front. Microbiol..

[102] Zheng Z., Tang J., Hu Y., Zhang W. (2022). Role of gut microbiota-derived signals in the regulation of gastrointestinal motility. Front. Med..

[103] Cai G.-X. (2011). Simotang enhances gastrointestinal motility, motilin and cholecystokinin expression in chronically stressed mice. World J. Gastroenterol..

[104] Singhal R., Ghadvaje G., Karra N., Gadde S.T., Chandra P., Voruganti B.K.T., Doddareddy N.P., Iftikhar S., Patel T. (2025). A narrative review on fecal microbiota transplantation routes in ulcerative colitis: Identifying the optimal approach across key parameters. Ann. Med. Surg..

[105] Wang S., Xu M., Wang W., Cao X., Piao M., Khan S., Yan F., Cao H., Wang B. (2016). Systematic Review: Adverse Events of Fecal Microbiota Transplantation. PLoS ONE.

[106] Bi Y., Cheng B., Zou B., Liu S., Cui Z. (2025). The current landscape of fecal microbiota transplantation in treating inflammatory bowel disease. Transl. Gastroenterol. Hepatol..

[107] Somineni H.K., Kugathasan S. (2019). The Microbiome in Patients with Inflammatory Diseases. Clin. Gastroenterol. Hepatol..

[108] Tchamani Piame L., Yako Y.Y. (2026). Impact of Single and Multi-Strain Probiotic Supplementation on Glycaemic Control in Type 2 Diabetic Patients: A Comparative Meta-Analysis. Appl. Biosci..

[109] Maslennikov R., Gosteeva E., Ananeva V., Korshunova L., Kravtsowa A., Poluektova E., Ulyanin A., Sigidaev A., Kikhasurova P., Ivashkin V. (2026). Strain-Specific Systematic Review with Meta-Analysis of Probiotics Efficacy in the Treatment of Irritable Bowel Syndrome. J. Clin. Med..

[110] Rahmannia M., Poudineh M., Mirzaei R., Aalipour M.A., Shahidi Bonjar A.H., Goudarzi M., Kheradmand A., Aslani H.R., Sadeghian M., Nasiri M.J. (2024). Strain-specific effects of probiotics on depression and anxiety: A meta-analysis. Gut Pathog..

[111] McFarland L.V., Karakan T., Karatas A. (2021). Strain-specific and outcome-specific efficacy of probiotics for the treatment of irritable bowel syndrome: A systematic review and meta-analysis. eClinicalMedicine.

[112] Lopes G., Campanari G., Barbalho S., Matias J., Lima V., Cressoni Araujo A., Goulart R., Detregiachi C., Haber J., Carvalho A. (2021). Effects of probiotics on inflammatory bowel disease: A systematic review. Jpn. J. Gastroenterol. Res..

[113] He X.-X., Li Y.-H., Yan P.-G., Meng X.-C., Chen C.-Y., Li K.-M., Li J.-N. (2021). Relationship between clinical features and intestinal microbiota in Chinese patients with ulcerative colitis. World J. Gastroenterol..

[114] Puvanasundram P., Chong C.M., Sabri S., Yusoff M.S., Karim M. (2021). Multi-strain probiotics: Functions, effectiveness and formulations for aquaculture applications. Aquac. Rep..

[115] Chapman C.M.C., Gibson G.R., Rowland I. (2012). In vitro evaluation of single- and multi-strain probiotics: Inter-species inhibition between probiotic strains, and inhibition of pathogens. Anaerobe.

[116] Kovačec E., Kraigher B., Podnar E., Lories B., Steenackers H., Mandic-Mulec I. (2024). *Bacillus subtilis* Intraspecies Interactions Shape Probiotic Activity Against *Salmonella* Typhimurium. Microb. Biotechnol..

[117] Wang X., Cheng Y., Huang J., Xu F., Jiang J., Nalinratana N., Jin L., Xue Y. (2026). Engineered probiotics for inflammatory bowel disease therapy: Mechanisms, delivery strategies, and precision medicine. Front. Microbiol..

[118] Jiang Y., Jiang S., Wang Z., Zhu P., Zhang J., Teng F., Huang S. (2026). Precise probiotic therapy: Advances, bottlenecks, and the road to microbiome-informed nutrition. Gut Microbes.

[119] Dore M.P., Bibbò S., Fresi G., Bassotti G., Pes G.M. (2019). Side Effects Associated with Probiotic Use in Adult Patients with Inflammatory Bowel Disease: A Systematic Review and Meta-Analysis of Randomized Controlled Trials. Nutrients.

[120] El-Saadony M.T., Saad A.M., Sitohy M., Alkafaas S.S., Dladla M., Ghosh S., Mohammed D.M., Ibrahim E.H., Fahmy M.A., Elkelish A. (2026). Probiotics and human health: Biological activities, nutritional aspects, immunomodulatory properties, applications, and future perspectives—A comprehensive review. Front. Immunol..

